# Differential brain iron deposition in Parkinson’s disease subtypes: a comparative quantitative susceptibility mapping study of tremor-dominant versus postural instability/gait difficulty phenotypes

**DOI:** 10.3389/fneur.2026.1788931

**Published:** 2026-03-24

**Authors:** Yiting Chen, Zengrui Zhang, Lifeng He, Ying Tan, Dan Wu, Xinyu Wang, Yang Li

**Affiliations:** 1 Clinical Nursing Office, Department of Nursing, Changzhou Hygiene Vocational Technology College, Changzhou, China; 2 Department of Neurology, Huzhou Central Hospital, Huzhou, China; 3 Department of Radiology, Huzhou Central Hospital, Huzhou, China

**Keywords:** dentate nucleus, iron deposition, motor subtypes, Parkinson’s disease, quantitative susceptibility mapping

## Abstract

**Introduction:**

This study used quantitative susceptibility mapping (QSM) to investigate brain iron deposition patterns in different motor subtypes of Parkinson’s disease (PD). We compared tremor-dominant (TD) and postural instability/gait difficulty (PIGD) patients, analyzed the association between iron deposition and core motor symptoms, and further explored its potential as an imaging feature associated with clinical subtypes.

**Methods:**

The study included 45 PD patients (25 TD-PD, 20 PIGD-PD) and 23 healthy controls. QSM values were measured in the bilateral caudate nucleus (CN), dentate nucleus (DN), globus pallidus (GP), putamen (PUT), red nucleus (RN), substantia nigra (SN), and thalamus (TH).

**Results:**

Compared to controls, both PD groups showed elevated iron levels in multiple brain regions. Notably, TD patients exhibited significantly higher magnetic susceptibility in the DN than both PIGD patients and controls, and DN susceptibility also showed a positive correlation with tremor severity in the overall PD cohort (rs = 0.339, *p* = 0.023). A combined model incorporating DN susceptibility and levodopa equivalent daily dose effectively distinguished TD from PIGD subtypes with an area under the curve of 0.898.

**Discussion:**

These findings suggest that the motor subtypes of Parkinson’s disease exhibit distinct brain iron distribution patterns, with the TD subtype showing increased DN iron deposition. This supports the role of the cerebello-thalamo-cortical (CTC) circuit in tremor and highlights dentate nucleus iron deposition as a potential imaging feature associated with the tremor-dominant phenotype.

## Introduction

Parkinson’s disease (PD) is a neurodegenerative disorder with significant clinical heterogeneity. Based on motor symptom presentation, it is commonly classified into tremor-dominant Parkinson’s disease (TD-PD), postural instability/gait difficulty Parkinson’s disease (PIGD-PD), and mixed subtypes (1). Different subtypes exhibit significant differences in disease progression rate, risk of cognitive decline, and treatment response (2–4), suggesting distinct underlying neuropathophysiological mechanisms. Tremor in TD-PD is considered to be closely related to the dysfunction of the cerebello-thalamo-cortical (CTC) circuit (5, 6), rather than simply attributed to the classical nigrostriatal dopamine depletion (7, 8). In contrast, the axial symptoms of the PIGD-PD are more closely related to the degeneration of the traditional nigrostriatal dopaminergic pathway and the involvement of a wider range of subcortical non-dopaminergic systems (9, 10).

Abnormal brain iron deposition is an important pathological feature of PD, closely related to oxidative stress, neuroinflammation, and dopaminergic neuron death (11, 12). Quantitative susceptibility mapping (QSM), as a magnetic resonance post-processing technology, can non-invasively and quantitatively evaluate the content of brain iron deposition, and provide a new tool for exploring the subtype-specific pathological changes of PD. Postmortem studies have confirmed a linear relationship between iron deposition in brain tissue and magnetic susceptibility values measured by QSM (13). Capitalizing on the superior quantitative capability of QSM in iron detection, current research is increasingly employing this technique to examine cerebral iron differences in PD. Previous studies have focused on the basal ganglia regions such as the substantia nigra and globus pallidus (14–16), while the iron deposition in the non-dopaminergic system-related nuclei such as the cerebellum has been relatively less concerned (17). The brain iron deposition pattern of PD motor subtypes is still not fully clear (18, 19). Therefore, this study aimed to use QSM to systematically compare iron deposition differences in multiple regions of interest (especially the cerebellar dentate nucleus) in the whole brain of TD-PD and PIGD-PD patients, analyze its association with specific symptoms like tremor and postural/gait impairment, and further examine the association between imaging and clinical indicators in relation to motor subtypes, in order to provide new imaging evidence for understanding the biological basis of PD clinical heterogeneity.

## Methods

### Study participants

This was a cross-sectional study. PD patients who visited the Department of Neurology at Huzhou Central Hospital from May 2024 to August 2025 were selected. Additionally, 23 age- and sex-matched healthy volunteers were included. Patient inclusion criteria: (1) Met the Movement Disorder Society (MDS) clinical diagnostic criteria for PD (2015) (20); (2) Were in the “ON” state and able to cooperate with MRI scanning and clinical assessment; (3) Had stable anti-PD medication regimens. Exclusion criteria: (1) Had other neurological diseases that could cause movement disorders or abnormal brain iron deposition; (2) Had contraindications for MRI examination; (3) Had incomplete clinical or imaging data. Healthy controls (HC) matched for age and sex were also recruited. All participants provided written informed consent.

### Clinical assessment and motor subtype classification

Demographic data were collected for all participants. Two neurologists assessed PD patients using the Unified Parkinson’s Disease Rating Scale (UPDRS), Hoehn & Yahr (H-Y) stage, and the Montreal Cognitive Assessment (MoCA). Levodopa equivalent daily dose (LEDD) was recorded according to the conversion factors (21, 22). Motor subtype classification: Based on the MDS recommended methodology, the tremor score and PIGD score were calculated based on the UPDRS-II and UPDRS-III sub-items. The tremor assessment comprised 8 items from UPDRS (each scored 0–4): (1) UPDRS-II item 16 (Tremor); (2) UPDRS-III item 20a (Facial resting tremor); (3) UPDRS-III item 20b (Right upper limb resting tremor); (4) UPDRS-III item 20c (Left upper limb resting tremor); (5) UPDRS-III item 20d (Right lower limb resting tremor); (6) UPDRS-III item 20e (Left lower limb resting tremor); (7) UPDRS-III item 21a (Right upper limb action or postural tremor); (8) UPDRS-III item 21b (Left upper limb action or postural tremor). The PIGD assessment comprised 5 items (each scored 0–4): (1) UPDRS-II item 13 (Falling); (2) UPDRS-II item 14 (Freezing); (3) UPDRS-II item 15 (Walking); (4) UPDRS-III item 29 (Gait); (5) UPDRS-III item 30 (Postural stability). The ratio (Tremor score / PIGD score) was calculated. A ratio ≥ 1.5 defined TD, a ratio ≤ 1 defined PIGD, and a ratio between 1 and 1.5 defined mixed type (mixed type was not included in intergroup comparisons in this study).

### MRI data acquisition and QSM processing

All scans were performed on a Siemens Magnetom Prisma 3.0 T MRI scanner using a 64-channel head coil. A multi-echo 3D gradient echo sequence was acquired for QSM reconstruction with the following parameters: repetition time (TR) = 46 ms; six equally spaced echoes with echo times (TEs) = 7.25, 12.0, 16.75, 21.5, 26.25, and 31.0 ms; flip angle = 20°; field of view (FOV) = 220 mm × 177 mm; matrix size = 256 × 256; slice thickness = 2 mm with no inter-slice gap; and 64 slices; resulting in a voxel size of 0.5 × 0.5 × 2 mm. The imaging volume covered the whole brain. The anisotropic voxel size was chosen as a trade-off between resolution and signal-to-noise ratio (SNR). Isotropic 0.5 × 0.5 × 0.5 mm resolution would have reduced voxel volume by a factor of four, leading to an unacceptable SNR decrease. The 0.5 mm in-plane resolution was prioritized to accurately delineate small deep brain nuclei, while the 2 mm slice thickness was considered acceptable as these nuclei are relatively thick in the superior–inferior direction and our analysis focused on whole-nucleus mean values. To reduce acquisition time, parallel imaging (mSENSE) was applied with an acceleration factor of 2. Acquisition time was 8 min and 7 s. The scanning slices were positioned parallel to the anterior commissure-posterior commissure (AC-PC) line, as determined by T1WI-MPRAGE images. During scanning, earplugs were provided, and participants were instructed to remain as still as possible. Foam padding was used to stabilize the head and minimize motion artifacts.

QSM image post-processing was performed using the MEDI toolbox (developed by Cornell University, United States) in MATLAB (23). Phase images from multi-echo GRE data were used to estimate the total field map via temporal unwrapping and weighted least-squares fitting, correcting residual spatial wraps with a magnitude-guided algorithm. A brain mask was generated using FSL BET and manually refined to exclude extracerebral tissues. The local field was separated from the background field using the projection onto dipole fields (PDF) method. Susceptibility maps were then computed with the MEDI algorithm, which applies morphological regularization. Finally, susceptibility values were referenced to white matter above the corpus callosum. Radiologists independently graded QSM image quality using the following grading system: 1 = very good (little or no artifact); 2 = good (visible artifacts); 3 = poor (considerable motion artifacts); 4 = very poor (significant motion artifacts), and 5 = non-diagnostic scan. Subjects with a consensus grading score higher than 2 were excluded from further analysis. In addition, routine axial T1-weighted imaging (T1WI), axial T2-weighted imaging (T2WI), axial diffusion-weighted imaging (DWI), and sagittal T2WI were acquired. These routine sequences were used to exclude other neurological disorders.

### ROI delineation and reliability

Regions of interest (ROIs) were manually drawn on QSM images using ITK-SNAP software^1^. ROIs were delineated slice-by-slice along the boundaries of deep brain nuclei, which appear as hyperintense regions on QSM images, reflecting elevated magnetic susceptibility values associated with abnormal iron deposition (13, 24). To ensure clear delineation of nuclear boundaries, all ROI drawings were performed at 4 × magnification, with reference to T1WI-MPRAGE and routine MRI sequences (25). A one-pixel inward offset was applied during contouring to ensure the delineated region was confined to the intranuclear tissue, while avoiding dilated perivascular spaces and physiological calcifications in the basal ganglia region. The following ROIs were delineated: caudate nucleus (CN), dentate nucleus (DN), globus pallidus (GP), putamen (PUT), red nucleus (RN), substantia nigra (SN), and thalamus (TH). The mean magnetic susceptibility value for each ROI was calculated and expressed in parts per billion (ppb).

All delineations were performed by radiologists blinded to clinical data. All radiologists received training in ROI delineation. To minimize intra-rater variability, each ROI was measured twice, and the average of the two measurements was used for analysis. The susceptibility value for each ROI was then calculated as the average of the bilateral structures (16, 26). The first radiologist delineated ROIs for all participants. Inter-rater reliability was assessed by comparing these measurements with those from a second blinded radiologist who independently re-delineated ROIs in 15 randomly selected participants. The intraclass correlation coefficient (ICC) was 0.948 (95% CI, 0.924–0.964).

### Statistical analysis

Statistical analysis was performed using SPSS 22.0. Normality of continuous variables was assessed using the Shapiro–Wilk test. Normally distributed continuous data are presented as mean ± standard deviation and compared using one-way ANOVA or independent samples *t*-test. Non-normally distributed data are presented as median (interquartile range) and compared using non-parametric tests. Categorical data were compared using the chi-square test.

To compare QSM values among the three groups, one-way analysis of covariance (ANCOVA) was performed with each brain region’s QSM values as the dependent variable, group as the fixed factor, and age and sex as covariates. *Post hoc* multiple comparisons were conducted using the Bonferroni method. An additional ANCOVA was also performed including age, sex, disease duration, H&Y stage, and LEDD as covariates to control for potential confounding by clinical differences.

Spearman rank correlation was used to analyze correlations between brain QSM values and clinical scores. These analyses were guided by *a priori* hypotheses derived from the literature, with two primary associations specified: the link between DN iron deposition and tremor, and the link between SN iron deposition and motor symptoms (27, 28). To account for multiple comparisons, Bonferroni correction was applied to these. All other correlations were considered exploratory.

To identify predictors for discriminating PIGD from TD subtypes, binary logistic regression analysis (forward: LR method) was performed with subtype (defined as PIGD = 0, TD = 1) as the dependent variable, and age, sex, disease duration, LEDD, and QSM values from brain regions showing intergroup differences as independent variables. To assess potential overfitting of the logistic regression model, we performed internal validation using bootstrapping with 1,000 resamples to calculate an optimism-corrected area under the curve (AUC).

Receiver operating characteristic (ROC) curves were constructed and the area under the curve (AUC) was calculated for three models: (1) a clinical model (age, sex, disease duration, LEDD), (2) an imaging-only model (DN QSM value alone), and (3) a combined model (clinical variables and DN QSM value). The DeLong test was used to compare the AUCs of the clinical model and the combined model. A *p*-value < 0.05 was considered statistically significant.

We performed a *post hoc* power analysis based on the primary outcome (DN difference between TD and PIGD) to assess the adequacy of our sample size.

## Results

### Comparison of demographic and clinical characteristics

Table 1 shows the demographic characteristics of all participants. A total of 25 TD patients, 20 PIGD patients, and 23 HCs were included. No significant differences were found in age or sex distribution among the three groups (*p* > 0.05). Comparison between TD and PIGD groups showed that the PIGD group had longer disease duration, higher H-Y stage, higher total UPDRS and Part I (mentation, behavior, and mood), Part II (activities of daily living), and Part III (motor examination) scores, larger LEDD, and a trend toward lower MoCA scores (*p* = 0.059). The core motor symptoms were successfully separated between subtypes: the TD group had higher tremor scores, while the PIGD group had significantly higher PIGD scores (all *p* < 0.05).

**Table 1:** Demographic and clinical data of the different subtype of PD patients and HC.

Variable	TD-PD (*n* = 25)	PIGD-PD (*n* = 20)	HC (*n* = 23)	Statistics	*P*
Age (years)	63.84 ± 9.73	65.80 ± 10.27	61.52 ± 6.90	*F* = 1.205	0.306
Sex (male/female)	9/16	12/8	11/12	*χ*^2^ = 2.577	0.276
Duration (years)	2(1.25, 5)	5(2, 10)	–	*U* = 157.000	0.033
LEDD (mg)	200.00(87.50, 325.00)	387.50(300.00, 506.25)	–	*U* = 87.500	<0.001
H-Y stage	2.0(1.5, 2.0)	3.0(2.125, 4.0)	–	*U* = 62.000	<0.001
UPDRS total	31.32 ± 13.20	58.25 ± 20.70	–	*t* = −5.303	<0.001
UPDRS-I	2.44 ± 1.50	4.15 ± 2.06	–	*t* = −3.220	0.002
UPDRS-II	7.88 ± 3.69	15.85 ± 7.32	–	*t* = −4.438	<0.001
UPDRS-III	18.00(10.50, 25.00)	30.50(24.25, 44.75)	–	*U* = 98.000	0.001
Tremor score	6.00(4.00, 9.50)	4.00(2.00, 8.00)	–	*U* = 159.500	0.038
PIGD score	2.00(1.00, 3.50)	8.00(6.00, 11.00)	–	*U* = 25.000	<0.001
MoCA	25.00(21.50, 27.00)	21.50(17.25, 25.50)	–	*U* = 167.500	0.059

Data are presented as mean ± standard deviation or median (interquartile range). UPDRS, Unified Parkinson’s Disease Rating Scale; H-Y, Hoehn & Yahr stage; LEDD, Levodopa Equivalent Daily Dose; MoCA, Montreal Cognitive Assessment.

### Comparison of brain magnetic susceptibility among groups

**Figure 1:**
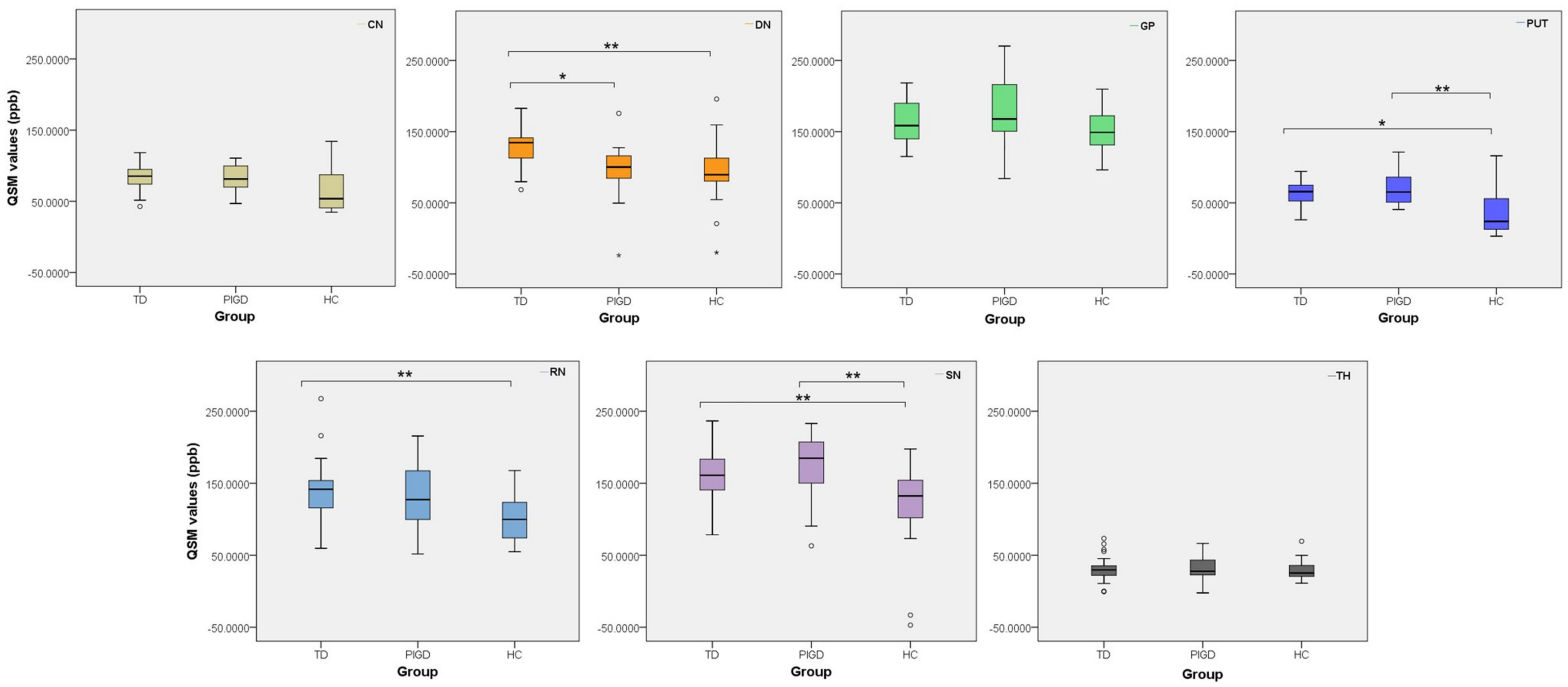
Boxplots of QSM values across all ROIs in TD, PIGD, and HC groups. Data are shown as box plots. *p < 0.05, **p < 0.01.

After controlling for age and sex, ANCOVA with Bonferroni correction revealed significant differences in QSM values among the three groups in several brain regions (Table 2). As shown in Figure 1, compared to the HC group, the TD group showed significantly higher magnetic susceptibility in DN, PUT, RN, and SN (all corrected *p* < 0.05). The PIGD group showed significantly higher magnetic susceptibility in PUT and SN (both corrected *p* < 0.05). Magnetic susceptibility in the DN was significantly higher in the TD group than in both the HC group (corrected *p* = 0.005) and the PIGD group (corrected *p* = 0.013). No difference was found in DN susceptibility between PIGD and HC groups. To control for potential confounding by clinical differences, we performed an additional ANCOVA including age, sex, disease duration, H&Y stage, and LEDD as covariates. The significant difference in DN magnetic susceptibility between TD and PIGD persisted (*F* = 9.266, *p* = 0.004, partial *η*^2^ = 0.200). In the SN, magnetic susceptibility was significantly higher in both PD subtypes compared to HC (corrected *p* < 0.05), but there was no difference between TD and PIGD. No significant differences were found among the three groups in CN, GP, or TH.

**Table 2:** Comparison of brain magnetic susceptibility among the three groups and *post hoc* tests.

Region	TD Group (*n* = 25)	PIGD Group (*n* = 20)	HCs (*n* = 23)	*F*	*P*	Partial*η*^2^	HC vs. TD	HC vs. PIGD	TD vs. PIGD
CN	85.28(71.81, 95.62)	81.35(67.45, 101.14)	53.64(38.82, 95.20)	3.382	0.040	0.097	n.s	n.s	n.s
DN	134.55(112.47, 144.40)	100.20(82.40, 116.23)	89.54(80.22, 114.89)	6.664	0.002	0.175	0.005	n.s	0.013
GP	164.89 ± 29.74	171.05 ± 47.41	153.53 ± 30.42	1.045	0.358	0.032	n.s	n.s	n.s
PUT	65.72(52.51, 75.09)	65.10(50.80, 86.48)	23.93(11.08, 70.94)	6.164	0.004	0.164	0.022	0.006	n.s
RN	139.99 ± 42.51	133.47 ± 43.11	102.34 ± 33.61	5.226	0.008	0.142	0.007	n.s	n.s
SN	160.93(140.54, 184.42)	184.62(144.82, 207.53)	132.33(101.25, 160.68)	7.773	0.001	0.198	0.005	0.002	n.s
TH	29.62(20.68, 36.99)	27.79(21.96, 43.42)	25.30(20.34, 36.63)	0.050	0.951	0.002	n.s	n.s	n.s

Data are presented as median (interquartile range) or mean ± standard deviation. QSM values are in ppb. CN, caudate nucleus; DN, dentate nucleus; GP, globus pallidus; PUT, putamen; RN, red nucleus; SN, substantia nigra; TH, thalamus. n.s., not significant (*P* > 0.05). The *p*-values presented for group comparisons are corrected significance levels.

### Correlation between brain magnetic susceptibility and clinical symptoms

**Figure 2:**
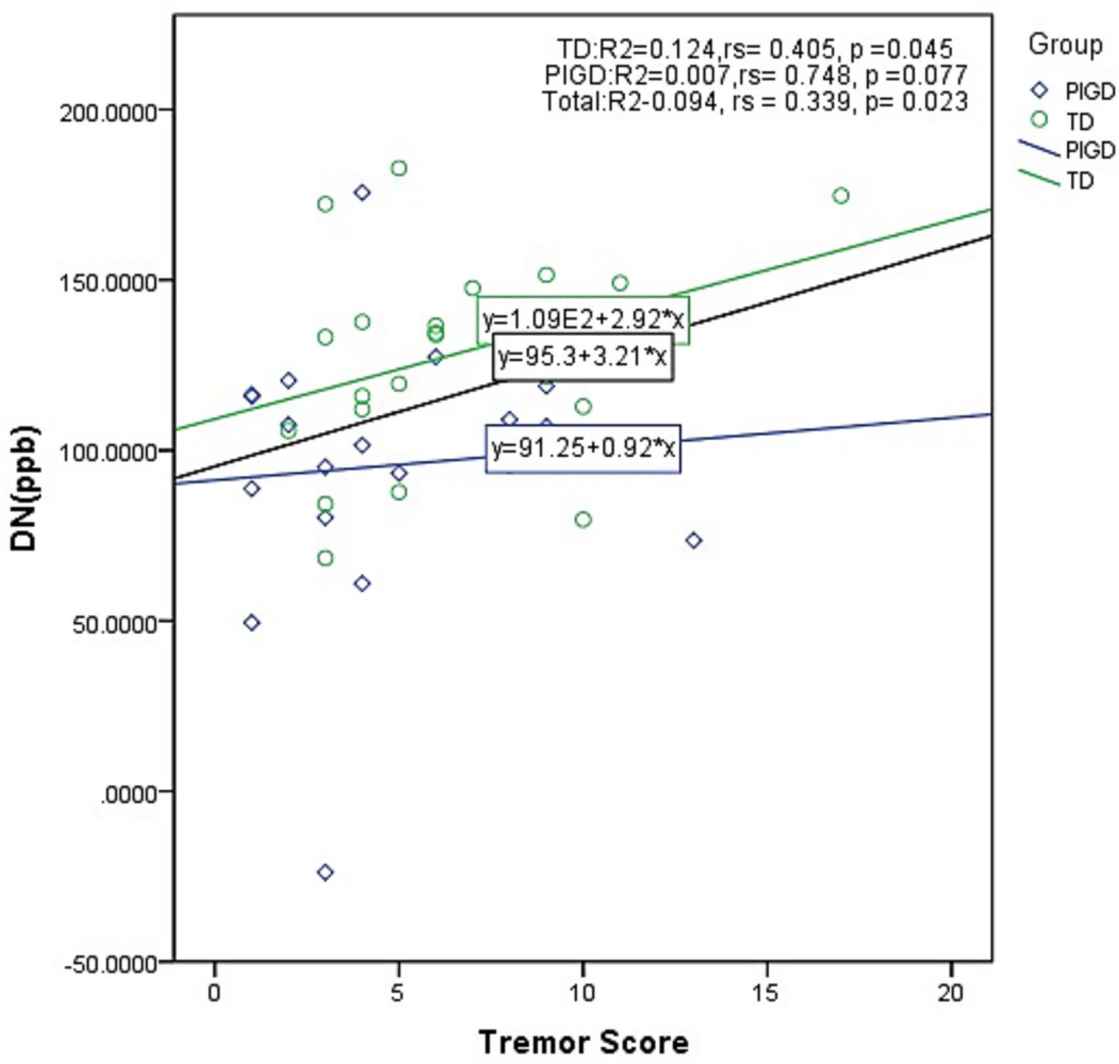
Scatter plot showing the correlation between DN magnetic susceptibility (QSM value) and tremor score. After Bonferroni correction, statistical significance was defined as *p* < 0.025.

Spearman correlation analysis indicated a positive association between DN QSM value and tremor score in the overall PD cohort (*n* = 45, rs = 0.339, *p* = 0.023), which met the prespecified significance threshold after Bonferroni correction (*p* < 0.025), as illustrated in Figure 2. This correlation was observed within the TD-PD group (rs = 0.405, *p* = 0.045), though it did not reach the corrected significance level, whereas no significant association was found in the PIGD-PD group (rs = 0.748, *p* = 0.077). Magnetic susceptibility in the SN showed a significant positive correlation with UPDRS-III motor score (rs = 0.362, *p* = 0.011), which also remained significant after Bonferroni correction. Exploratory analyses of other brain regions (CN, GP, PUT, RN, and TH) revealed no significant correlations with tremor or other clinical measures (all *p* > 0.05).

### Subtype discrimination model based on logistic regression

**Figure 3:**
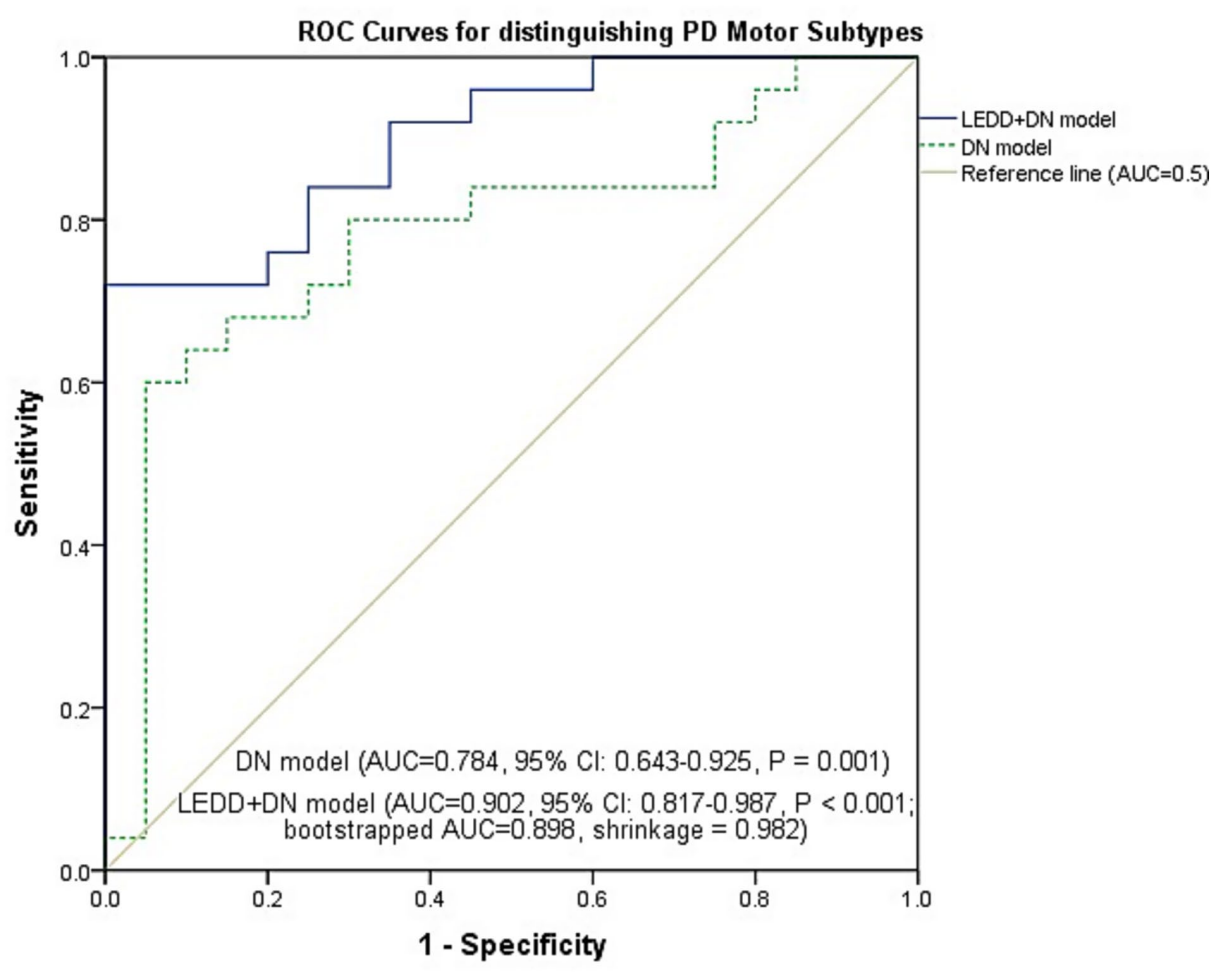
ROC curves for the imaging-only model (DN QSM value alone) and the combined model (LEDD and DN QSM value) in discriminating TD from PIGD subtypes.

Binary logistic regression with forward stepwise selection (likelihood ratio) was performed with subtype (TD = 1, PIGD = 0) as the dependent variable, and age, sex, disease duration, LEDD, and DN QSM value as independent variables. The final model included two independent predictors: LEDD and DN QSM value, as detailed in Table 3. The OR for LEDD was less than 1 (OR = 0.989, *p* = 0.002), indicating that higher LEDD was associated with the PIGD subtype. Conversely, the OR for DN QSM value was greater than 1 (OR = 1.039, *p* = 0.014), indicating that higher DN magnetic susceptibility was associated with the TD subtype. The model was statistically significant (*χ*^2^ = 26.638, *p* < 0.001), with a Nagelkerke *R*^2^ of 0.598. The overall prediction accuracy was 75.6%, with a sensitivity of 76.0% and specificity of 75.0%. We further evaluated the discriminative ability of two models using ROC analysis: an imaging-only model (DN QSM value alone) and a combined model (LEDD and DN QSM value). As shown in Figure 3, the imaging-only model yielded an AUC of 0.784 (95% CI, 0.643–0.925, *p* = 0.001), indicating moderate ability to distinguish TD from PIGD subtypes. The combined model demonstrated superior discriminative performance, with an AUC of 0.902 (95% CI, 0.817–0.987, *p* < 0.001). To assess potential overfitting, we performed internal validation using bootstrapping. The optimism-corrected AUC for the combined model was 0.898, with a shrinkage coefficient of 0.982, indicating minimal overfitting and good model stability.

**Table 3:** Multivariate logistic regression analysis for discriminating PIGD from TD subtypes.

Variable	*B*	S. E.	Wald	*P*	OR	95% CI
LEDD	−0.011	0.004	9.168	0.002	0.989	0.982–0.996
DN	0.038	0.015	6.061	0.014	1.039	1.008–1.071
Constant	−0.644	1.893	0.116	0.734	0.525	–

Model *χ*^2^ = 26.638, *P* < 0.001; Nagelkerke *R*^2^ = 0.598; Overall accuracy = 75.6%; Sensitivity = 76.0%; Specificity = 75.0%.

### ROC curve analysis of subtype discrimination models

**Figure 4:**
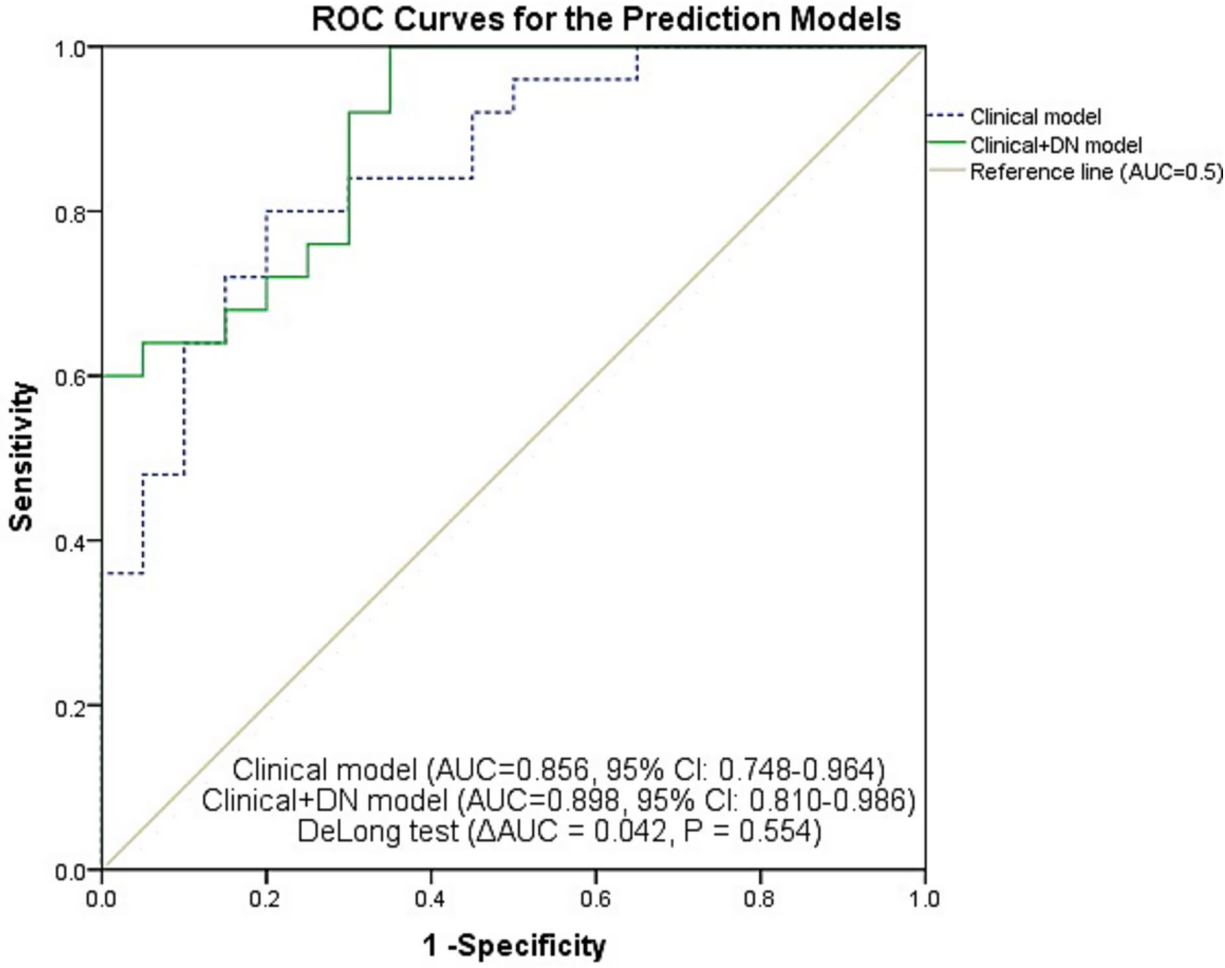
ROC curves for the clinical model (age, sex, disease duration, LEDD) and the combined model (clinical variables + DN QSM) in discriminating TD from PIGD subtypes.

To further evaluate discrimination performance, ROC curves were plotted for a clinical model (age, sex, disease duration, LEDD) and a clinical plus imaging model (age, sex, disease duration, LEDD, DN QSM value), as shown in Figure 4. The clinical model achieved an AUC of 0.856 (95% CI, 0.748–0.964), with an optimal cutoff of 0.545 based on the Youden index, corresponding to a sensitivity of 80.0% and a specificity of 80.0%. The combined model yielded a higher AUC of 0.898 (95% CI, 0.810–0.986), and its optimal cutoff was 0.241, with a sensitivity of 100.0% and a specificity of 65.0%. However, the DeLong test showed no significant difference between the two AUCs (ΔAUC = 0.042, *p* = 0.554), suggesting that the addition of DN iron deposition did not significantly improve discriminative performance beyond clinical variables alone.

### *Post hoc* power analysis

Based on the primary outcome of DN magnetic susceptibility difference between TD and PIGD groups, a *post hoc* power analysis was conducted. With an effect size of Cohen’s *d* = 0.99, alpha = 0.05, and power = 0.80, the required sample size per group was 18. Our actual sample (TD = 25, PIGD = 20) exceeds this, indicating sufficient power for the main comparison.

## Discussion

This study utilized Quantitative Susceptibility Mapping (QSM) to compare differences in brain iron deposition between motor subtypes of Parkinson’s disease. In this study, we used QSM to measure magnetic susceptibility, which is closely correlated with iron deposition in deep brain nuclei (13). Our findings revealed that significant differences in both clinical presentation and cerebellar iron deposition between tremor-dominant (TD) and postural instability/gait difficulty (PIGD) patients. Notably, magnetic susceptibility levels in the cerebellar dentate nucleus (DN) was closely related to tremor severity. This provides imaging evidence for the clinical heterogeneity of Parkinson’s disease (PD) and suggests that QSM may serve as a useful tool for characterizing subtype-specific brain iron patterns.

Clinically, PIGD-PD patients exhibited longer disease duration, higher H-Y stage, more severe non-motor (e.g., neuropsychiatric) and motor symptoms, and significantly higher levodopa equivalent daily dose (LEDD). This is consistent with previous studies (29, 30) reporting that the PIGD subtype is associated with faster disease progression, more severe dopaminergic deficit, and poorer prognosis. Aleksovski et al. (29) observed a greater susceptibility to cognitive decline in PIGD patients during a four-year longitudinal follow-up study. Although no significant difference was found in the present study, intergroup comparisons of cognitive levels also revealed a declining trend in PIGD patients (*p* = 0.059). In contrast, TD-PD patients exhibited a relatively slower disease progression (31, 32) and demonstrate greater heterogeneity in their response to dopaminergic medications (33).

This study found that DN iron deposition in TD patients was significantly higher than in both healthy controls and the PIGD group (both *p* < 0.05). Correlation analysis further revealed a significant positive correlation between DN QSM value and tremor scores in the overall PD group (rs = 0.339, *p* = 0.023), and this association was also observed within the TD-PD group (rs = 0.405, *p* = 0.045), but did not reach statistical significant in the PIGD-PD group. Although the TD-PD group correlation did not survive Bonferroni correction for multiple comparisons (significance threshold set at *p* < 0.025), likely due to the reduced sample size, the moderate effect size (*r* = 0.405) and its consistency with the overall finding suggest biological plausibility. These results are in line with previous studies and underscore the need for further investigation in larger cohorts. Zhang et al. (26) noted that the DN is one of the brain nuclei with high iron deposition and that its QSM value was significantly elevated only in TD patients. Guan et al. (34) also observed that iron deposition in the DN and red nucleus of TD patients correlated with tremor severity. Chen et al. (35) explicitly reported lower DN iron deposition in PIGD-PD compared to TD-PD patients, hypothesizing that the cerebellum may be in a state of reduced activity within the PIGD subtype. This contrasts with the hypothesis of excessive activity in the CTC circuit within the TD subtype, highlighting the cerebellum’s central role in motor regulation and postural stability. Collectively, this evidence suggests that iron deposition in the DN, the sole output nucleus of the cerebellum (36), is a subtype-associated imaging feature that may aid in characterizing TD-PD from PIGD-PD.

An intriguing finding of this study is that TD patients exhibited higher DN iron deposition than PIGD patients, despite having milder overall disease burden as indicated by shorter disease duration, lower H&Y stages, and lower LEDD. This seemingly paradoxical finding suggests that DN involvement may be specifically linked to tremor pathophysiology rather than simply reflecting global disease progression. Several lines of evidence support this interpretation. First, DN iron deposition correlated with tremor severity in the overall PD cohort and within the TD subgroup, but showed no significant correlation with disease duration or H&Y stage. Second, previous studies have similarly reported elevated DN iron deposition in TD patients without corresponding increases in other markers of disease severity (26, 35). Third, the cerebellum and its output pathways have been increasingly recognized as key nodes in tremor-generating networks, with functional imaging studies demonstrating hypermetabolism and altered connectivity in the CTC circuit in TD-PD (5, 6, 37). Taken together, these observations support the notion that DN iron deposition in TD-PD reflects a circuit-specific vulnerability rather than a nonspecific consequence of advancing disease.

Building on this, we constructed a discrimination model using binary logistic regression. The final stepwise regression model retained LEDD and DN QSM value as independent predictors, showing that higher DN iron deposition was an independent predictor associated with the TD subtype, separate from clinical variables like LEDD. This model demonstrated good discriminative ability with an AUC of 0.902 (95% CI, 0.817–0.987). Bootstrap internal validation (1,000 resamples) confirmed its stability, yielding an optimism-corrected AUC of 0.898 and a shrinkage coefficient of 0.982, indicating minimal overfitting. The imaging-only model (DN QSM alone) demonstrated moderate discriminative ability with an AUC of 0.784 (95% CI, 0.643–0.925, *p* = 0.001), indicating that DN susceptibility alone can distinguish TD from PIGD subtypes to some extent. To further assess the incremental value of DN iron deposition over clinical information, we compared a clinical model (age, sex, disease duration, LEDD) with a combined model that additionally included DN QSM value. The clinical model achieved an AUC of 0.856 (95% CI, 0.748–0.964), while the combined model yielded an AUC of 0.898 (95% CI, 0.810–0.986). The DeLong test revealed no significant difference between the two AUCs (ΔAUC = 0.042, *p* = 0.554), suggesting that the information provided by DN QSM value largely overlaps with that of clinical variables. It is important to note that these clinical variables are not part of the formal definition of motor subtypes, which is based solely on the tremor/PIGD ratio derived from UPDRS items. Hence, including them in the model does not introduce circularity; rather, it allows us to test whether DN iron deposition provides information beyond these routine clinical indicators. Although LEDD is influenced by disease severity and subtype classification, its inclusion in the model serves as a conservative adjustment to test whether DN iron deposition provides independent information beyond this clinically relevant variable. The fact that magnetic susceptibility in the DN remained a significant predictor after adjusting for LEDD strengthens the validity of this association. Nevertheless, the independent association of DN iron deposition with the TD subtype supports the involvement of the CTC circuit in tremor pathophysiology. The overlap between DN iron deposition and clinical variables such as LEDD is biologically plausible: dentate nucleus pathology may contribute to the clinical phenotype that influences medication requirements, manifesting downstream as differential clinical features and medication needs. This interpretation aligns with the view that PD motor subtypes arise from distinct neural circuit vulnerabilities.

In contrast, iron deposition in the substantia nigra (SN) was significantly higher in both TD and PIGD groups compared to healthy controls, but no statistical difference existed between the two PD subtypes. This suggests that SN iron abnormality is a common pathological feature across PD subtypes, primarily reflecting dopaminergic neuron degeneration, but its degree is insufficient to distinguish between movement subtypes. The currently accepted pathological mechanism of PD is primarily located in the nigrostriatal dopaminergic system (38), and abnormal SN iron deposition is widely recognized as a key pathological feature (15, 39, 40). However, research has confirmed that pathological changes associated with *α*-synuclein are also present in the cerebellum of Parkinson’s disease patients (41–43). Numerous studies have detected high levels of dopamine and dopaminergic receptor subtypes in the human cerebellum (44, 45), indicating that the cerebellum plays a significant role in the pathogenesis of PD, particularly in the development of tremor. The dissociation between DN and SN iron deposition patterns suggests that the clinical heterogeneity of PD may stem from differential vulnerability of distinct neural circuits to pathological insult: TD symptoms might be more driven by dysfunction in cerebellar and cerebello-thalamo-cortical (CTC) circuit (5, 6), while PIGD symptoms may be more related to involvement of the nigrostriatal pathway and striatal-thalamic-cortical (STC) circuit (46).

Mechanistically, abnormal iron deposition in the DN, the primary output nucleus of the cerebellum, may reflect neuronal metabolic disturbance in this region due to processes like oxidative stress and neuroinflammation. Such structural changes could interfere with the normal function and rhythmic regulation of the CTC circuit, thereby contributing to the generation and maintenance of tremor. Functional imaging studies also suggest differences in brain network connectivity patterns between TD-PD and PIGD-PD patients, particularly in circuits involving the cerebellum and sensorimotor networks (38, 47–50). In recent years, the “Finger-Switch-Dimmer” model has further complemented the research of Duval et al. (51), building upon the work of Helmich et al. (52), suggesting that PD tremor may originate in the basal ganglia (the finger), particularly the medial pallidum. The thalamus generates tremor oscillations (the switch), which are amplified and modulated via the CTC circuit, functioning as a “dimmer” (8, 53–55). The correlation between DN iron deposition and tremor found in our study, along with the specific alteration of the DN in the TD subtype, provides direct quantitative imaging support for this hypothesis, further emphasizing the key role of the cerebellum in PD tremor mechanisms.

These findings may have several clinical implications. First, the independent association between DN iron deposition and the TD subtype, even after adjusting for clinical confounders, suggests that QSM may serve as an adjunctive imaging biomarker for subtype characterization. This may aid in patient stratification for clinical trials and contribute to a more nuanced understanding of individual prognosis. Second, the dissociation between DN and SN involvement supports the notion that PD motor subtypes arise from distinct neural circuit pathologies. For the TD subtype, modulating CTC circuit function or exploring neuroprotective strategies aimed at reducing iron deposition or oxidative stress in cerebellar nuclei might more precisely alleviate tremor. For the PIGD subtype, the focus may remain on optimizing dopaminergic replacement, combined therapy targeting non-dopaminergic systems, and proactive gait rehabilitation. These circuit-based insights may ultimately guide more personalized therapeutic approaches. However, given the cross-sectional design, longitudinal studies are needed to determine whether DN iron deposition precedes or follows the clinical manifestation of tremor.

### Limitations

This study has certain limitations. First, Cross-sectional designs cannot establish causal relationships between iron deposition and subtype characteristics, nor can they depict their dynamic evolutionary trajectories. Second, while the overall sample size was determined to be adequate for the primary comparison between TD and PIGD groups based on a *post hoc* power analysis, it was limited for some secondary explorations. In particular, the relatively small number of PIGD patients (n = 20) in subgroup correlation analyses (e.g., within the PIGD group) and logistic regression modeling may have reduced the statistical power to detect weaker associations and increased the risk of model overfitting, despite the use of stepwise selection and internal validation. Therefore, findings from these secondary analyses should be interpreted with caution and warrant validation in larger, independent cohorts. Third, Future large-sample, longitudinal studies are needed to validate the temporal changes in DN iron deposition and its relationship with clinical symptom progression. Concurrently, integrated analysis combining multimodal neuroimaging, genetics, and humoral biomarkers will help build a more comprehensive and precise PD subtype classification and prediction system.

## Conclusion

In summary, this QSM study revealed that different motor subtypes of Parkinson’s disease correspond to distinct brain iron deposition patterns. A characteristic finding in the tremor-dominant (TD) subtype is increased iron deposition in the cerebellar dentate nucleus, which correlates with tremor symptoms. This association supports the involvement of the cerebello-thalamo-cortical (CTC) circuit in tremor pathophysiology, and highlights dentate nucleus iron as a potential imaging feature linked to this specific motor phenotype. The postural instability/gait difficulty (PIGD) subtype is associated with greater overall disease severity and higher dopaminergic medication requirements. Dentate nucleus iron content may serve as a useful imaging feature associated with the TD subtype. These findings deepen the understanding of the pathological basis of PD clinical heterogeneity from the perspective of brain iron metabolism and provide new insights into the neural circuits underlying different motor phenotypes.

## Data Availability

The original contributions presented in the study are included in the article/supplementary material, further inquiries can be directed to the corresponding author.

## Footnotes

1
https://sourceforge.net/projects/itk-snap/
